# Diversity of Mycobiota Associated with the Cereal Cyst Nematode *Heterodera filipjevi* Originating from Some Localities of the Pannonian Plain in Serbia

**DOI:** 10.3390/biology10040283

**Published:** 2021-04-01

**Authors:** Violeta Oro, Rade Stanisavljevic, Bogdan Nikolic, Marijenka Tabakovic, Mile Secanski, Solveig Tosi

**Affiliations:** 1 Department of Plant Diseases, Institute for Plant Protection and Environment, 11000 Belgrade, Serbia; stanisavljevicrade@gmail.com (R.S.); bogdannik@mail2world.com (B.N.); 2 Agroecology and Cropping Practices Group, Maize Research Institute “Zemun Polje”, 11000 Belgrade, Serbia; mtabakovic@mrizp.rs (M.T.); msecanski@mrizp.rs (M.S.); 3 Mycology Laboratory, Department of Earth & Environmental Sciences, University of Pavia, 27100 Pavia, Italy; solveig.tosi@unipv.it

**Keywords:** fungi, cereal cyst nematode, phylogeny, Pannonia

## Abstract

**Simple Summary:**

*Heterodera filipjevi*, the cereal cyst nematode, is one of the most globally recognized and economically important nematodes on wheat. As some other cyst nematodes that are plant root parasites, the cysts of *H. filipjevi* survive in soil for years and shelter a large number of microbes. The aims of this study were to investigate the diversity of mycobiota associated with the cereal cyst nematode *H. filipjevi*, to infer phylogenetic relationships of the found mycobiota, and to explore the ecological connection between fungi and the field history, including the potential of fungi in bioremediation and the production of novel bioactive compounds. The study showed that the fungal species associated with the *H. filipjevi* cysts belong to diverse phyla, including Ascomycota, Basidiomycota, and Mucoromycota. The members of Ascomycota (*Fusarium avenaceum*, *Sarocladium kiliense*, *Setophoma terrestris*) are plant parasites, indicating that crops were host plants for fungal infection of recent origin. The members of Basidiomycota (*Bjerkandera adusta*, *Cerrena unicolor*, *Trametes hirsuta*, etc.) are wood-decay fungi, the presence of which in agricultural soil indicates that forests were the preceding plants.

**Abstract:**

Cereals, particularly wheat, are staple food of the people from the Balkans, dating back to the Neolithic age. In Serbia, cereals are predominantly grown in its northern part between 44° and 45.5° N of the Pannonian Plain. One of the most economically important nematodes on wheat is the cereal cyst nematode, *Heterodera filipjevi.* Cysts of *H. filipjevi* survive in soil for years and shelter a large number of microorganisms. The aims of this study were to investigate the diversity of mycobiota associated with the cereal cyst nematode *H. filipjevi*, to infer phylogenetic relationships of the found mycobiota, and to explore the ecological connection between fungi and the field history, including the potential of fungi in bioremediation and the production of novel bioactive compounds. Cysts were isolated from soil samples with a Spears apparatus and collected on a 150-µm sieve. The cysts were placed on potato dextrose agar, and maintained for two weeks at 27°C. Following fungal isolation and colony growing, the fungal DNA was extracted, the ITS region was amplified, and PCR products were sequenced. The study showed that the isolated fungal species belong to diverse phyla, including Ascomycota, Basidiomycota, and Mucoromycota. Ascomycota is represented by the families Clavicipitaceae, Sarocladiaceae, Nectriaceae, and Phaeosphaeriaceae. Basidiomycota is represented by the families Cerrenaceae, Polyporaceae, Phanerochaetaceae, and Meruliaceae, and the order Cantharellales. The family Mortierellaceae represents Mucoromycota. The members of Ascomycota and Basidiomycota both depict the field history. Ascomycota indicate the fungal infection is of recent origin, while Basidiomycota point toward the preceding host plants, enabling the plant field colonization history to be traced chronologically.

## 1. Introduction

Growing cereals in the Balkans in the region around the Danube and Pannonian Basin dates back to the Neolithic age [1]. Cereals, particularly wheat, were and still are a staple food of the people from this region. In Serbia, cereals are predominantly grown in its northern part between 44° and 45.5° N of the Pannonian Plain. *Heterodera filipjevi*, also known as the wheat cyst nematode, is one of three main species of the Cereal Cyst Nematode complex, which are the most globally recognized and economically important nematodes on wheat [2]. Cysts are swollen females containing eggs and juveniles, while males have a short life span and they are rarely found in soil. As some other cyst nematodes, the cysts of *H. filipjevi* survive in soil for years and shelter a large number of microorganisms, the presence of which can lead to cyst death and population decline [3]. Natural openings (e.g., the mouth, anus, and the vulva) are the entrances of fungal cyst invasion [4]. Fungi hold important positions among the microorganisms by their antagonistic behavior, and some of them have shown great potential as biocontrol agents [5].

The fungal antagonists of nematodes generally comprise four groups, the nematode-trapping fungi, endoparasites, parasites of nematode cysts and eggs, and fungi producing toxic metabolites [6]. *Arthrobotrys* sp. forms traps that take many forms including sticky knobs, sticky nets, or rings. During the physical contact between nematodes and fungi, the ring expands rapidly crushing the prey, which is then digested within hours. It is well recognized that species of the Basidiomycota are the best degraders of wood. Most significantly, the genus *Nematoctonus* (syn. *Hohenbuehelia*) produces both cellulases and ligninases, the principal enzymes used by wood-decay fungi [7]. The obligate endoparasitic fungus *Meria coniospora* (syn. *Drechmeria coniospora*) lives its entire vegetative life within infected nematodes. Conidia of *M. coniospora* infect the nematode *Panagrellus redivivus* mainly in the mouth region [8]. *Nematophthora gynophila* causes total destruction of *Heterodera avenae* cysts in less than seven days [9]. *Acremonium strictum* (syn. *Sarocladium strictum*) and *Fusarium oxysporum* are the main parasites in eggs of *H. schachtii* [10]. The most frequent egg parasites that developed in eggs of *H. avenae* were *Verticillium* spp. (including *V. chlamydosporium*, syn. *Pochonia chlamydosporia*) and *Paecilomyces carneus* (syn. *Metarhizium carneum*) [11]. Combined application of *Paecilomyces lilacinus* (syn. *Purpureocillium lilacinum*) and *Monacrosporium lysipagum* (syn. *Dactylella lysipaga*) reduced 65% of *H. avenae* cysts [12]. Metabarcoding of the fungal classes isolated from the soybean cyst nematode *H. glycines* revealedfOrbiliomycetes, Dothideomycetes, Eurotiomycetes, Sordariomycetes, Leotiomycetes, and Pezizomycetes in Ascomycota, the Agaricomycetes in Basidiomycota, the Glomeromycetes and Mortierellomycetes in Mucoromycota [13]. *Fusarium oxysporum* produced culture filtrates toxic to nematodes. These metabolites reduced *Meloidogyne incognita* mobility within 10 min of exposure. The second-stage juveniles were initially inactivated within a few minutes of exposure, but with exposure of 24 h, 100% of the juveniles were dead [14]. Flavipin, a low molecular weight metabolite of the fungus *Chaetomium globosum* is responsible for most of the nematode-antagonistic activity [15].

The aims of this study were to investigate the diversity of mycobiota associated with the cereal cyst nematode *H. filipjevi*, to infer phylogenetic relationships of the found mycobiota, based on the Maximum likelihood and Bayesian phylogeny of the internal transcribed spacer sequence region (ITS), and to explore the ecological connection between fungi and the field history, including the potential of fungi in bioremediation and the production of novel bioactive compounds.

## 2. Materials and Methods

### 2.1. Isolation of Nematodes and Fungi

The cysts of *H. filipjevi* were found in the following localities of the Pannonian Plain: Ada (45°48′ N; 20°07′ E), Dobric (44°41′ N; 19°34′ E), Feketic (45°38′ N; 19°39′ E), Indjija (45°03′ N; 20°05′ E), Kula (45°36′ N; 19°29′ E), Mol (45°45′ N; 20°05′ E), and Veliki Radinci (45°02′ N; 19°39′ E). Cereals, i.e., wheat, were in rotation with sugar beet and maize. After wheat harvest, 50 soil subsamples/hectare were taken to form one kilogram of a mixed sample [16]. Using the elutriator of Spears [17], which can process both dry and wet soil samples, cysts were separated and collected on a 150-µm sieve. Cysts of the wheat nematode were morphologically identified, and then the species identity was confirmed by molecular methods. *Heterodera filipjevi* was found in a single population in all localities, except in Kula, where it was detected in a mixed population with *H. schachtii*. Thirty randomly selected cysts from each location were sterilized applying the procedure of Heungens et al. [18]. The cysts were placed on potato dextrose agar (PDA) containing antibiotics (bensylpenicillin K+bensylpenicillin-procaine, 200.000 i.u. +600.000 i.u., 200 mg/L) and maintained for two weeks at 27 °C. After emergence of fungi on PDA, the fungi were subcultured by aseptically transferring small pieces of mycelium or spores to fresh PDA [19] using a dissecting microscope and pure cultures of each isolates were maintained in PDA slant tubes at room temperature. Air-dried cysts [20] were sputter-coated with gold, and viewed with a Jeol JSM-6460 LV scanning electron microscope to examine fungal cyst colonization.

### 2.2. Molecular Study

The extraction of DNA from the fungi (and nematodes) was performed with the DNeasy Blood and Tissue Kit (Qiagen) according to the manufacturer’s procedure, using approximately 10 mm of fungal tissue scraped from freshly-grown mycelium or one cyst. The ITS1-5.8S-ITS2 region was used for sequencing of fungi and the same protocol and primers were used for nematodes. Amplification of the internal transcribed spacer (ITS) region was performed by using 2234C and 3126T primers [21]. The PCR reaction mixture consisted of 1× PCR reaction buffer, 0.2 µM of forward and reverse primers, 200 µM dNTPs, 0.1 U/µL Taq Fermentas, 1 µL of DNA template, and nuclease-free water to a total volume of 20 µL. The protocol for the PCR reaction was carried out with the following parameters: 95 °C for 120 s followed by 35 cycles consisting of 95 °C for 30 s, 55 °C for 30 s, and 72 °C for 90 s. The reaction mixture was then incubated at 72 °C for 3 min [22]. Following the purification and sequencing of the obtained PCR products, the sequences were deposited in The National Center for Biotechnology Information nucleotide database (USA), under accession numbers MW485436-MW485447. Phylogenetic analyses were carried out employing Maximum likelihood (ML) and Bayesian inference (BI), generated by PhyML 3.1 [23], and MrBayes 3.1.2 [24] programs, respectively. The sequence alignment was performed with the ClustalW module of Mega 4 [25]. The Maximum likelihood dendrogram was obtained with the General Time Reversible model (GTR), invariable sites and gamma distribution (GTR+I+G). The consensus tree with 50% majority rule obtained by Bayesian inference was created by 1.6 × 10^6^ generations of Markov Chain Monte Carlo, sampling each 100th generation and “burnin” function of 20%. The nucleotide evolution model was the GTR+I+G as well. Branch supports higher than 70% were shown next to the node. *Heterodera filipjevi* and *H. avenae* served as outgroups.

## 3. Results and Discussion

**Figure 1:**
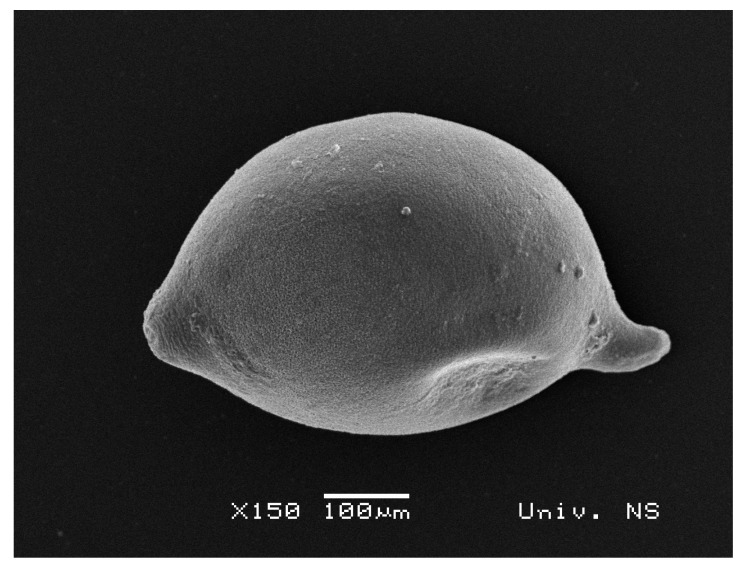
Scanning electron microscopy of *H. filipjevi* cyst.

**Figure 2:**
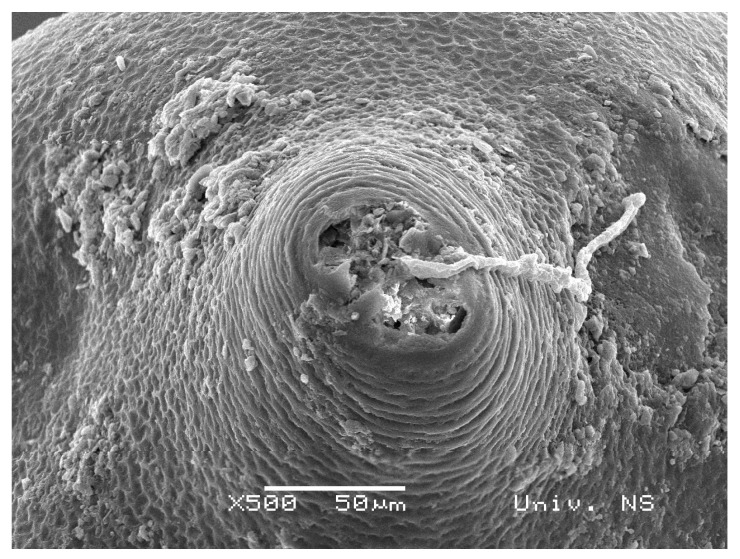
Scanning electron microscopy showing the vulval area of a cyst and a fungal hypha.

Cysts i.e., swollen females, containing eggs and second-stage juveniles, represent an ideal growth medium for diverse microorganisms. By its shape (Figure 1), a cyst is a closed “micro system” with proteins, lipids, chitin, carbohydrates, and other organic compounds [26] that can be utilized by microorganisms as a nutrition source. The fungal cyst colonization often starts via natural openings e.g., the vulva, located in a conical posterior part of the female body (Figure 2).

**Figure 3:**
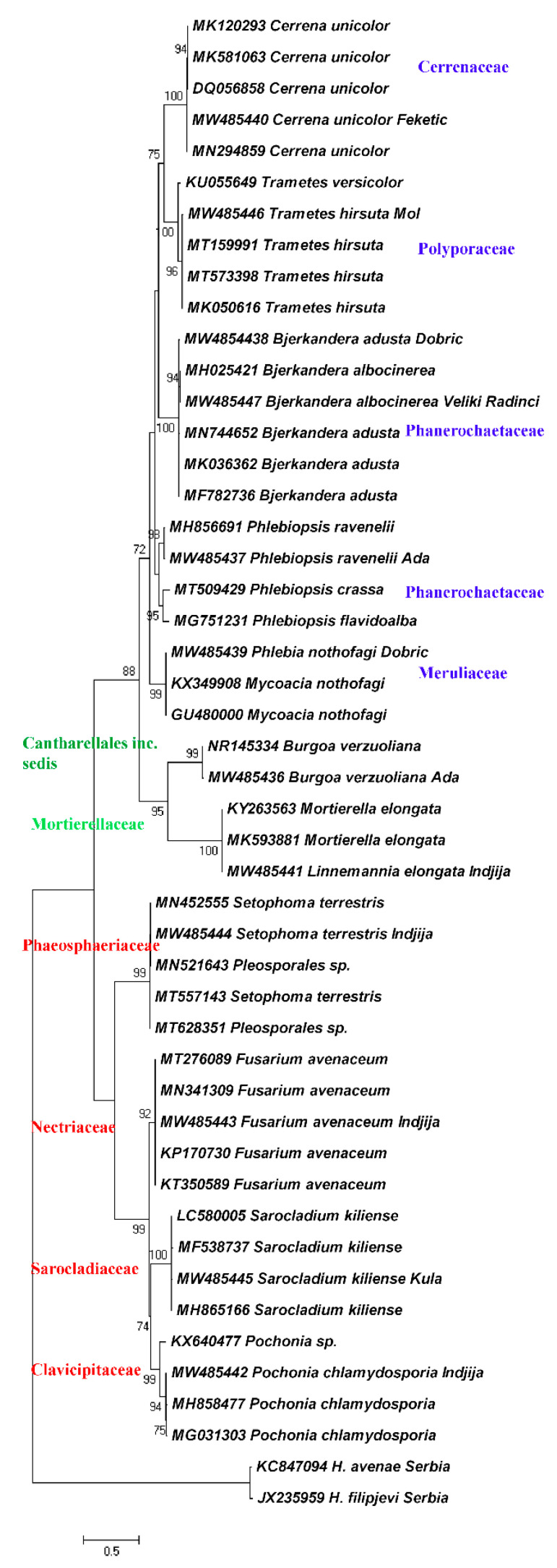
Maximum likelihood dendrogram of mycobiota associated with *H. filipjevi* cysts based on the ITS1-5.8S-ITS2 sequences and the GTR+I+G nucleotide evolution model.

**Figure 4:**
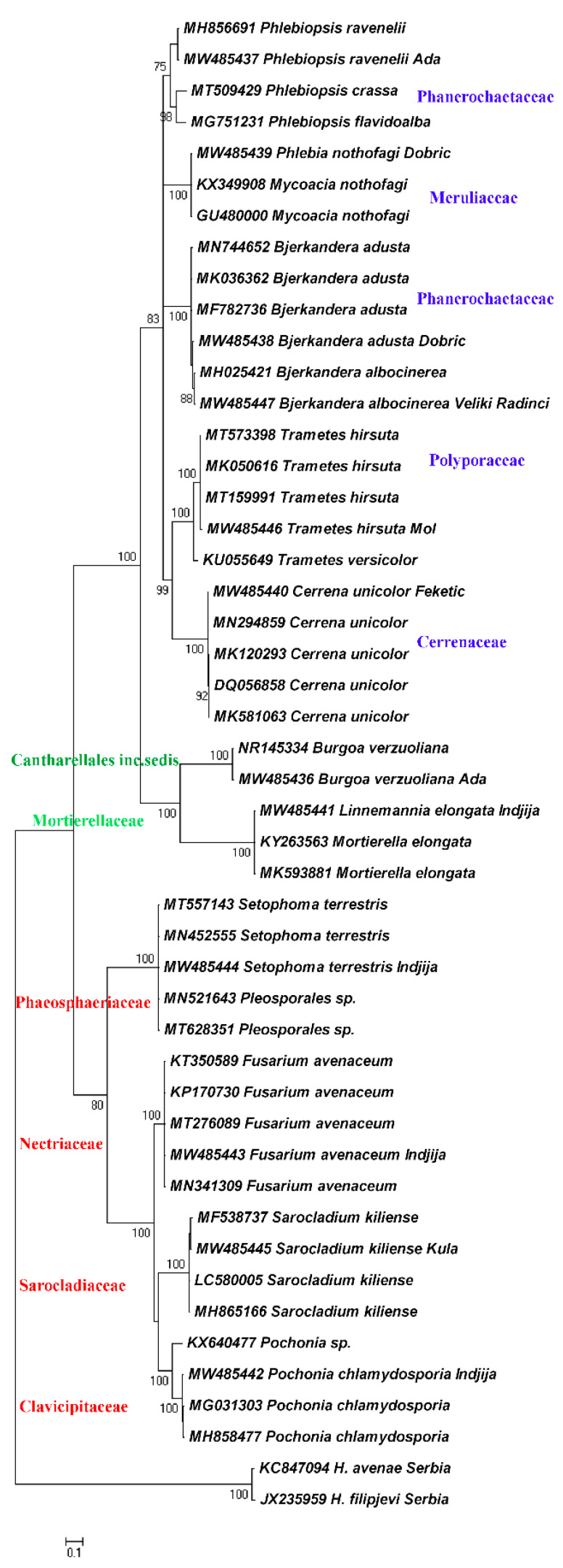
Bayesian dendrogram of mycobiota associated with *H. filipjevi* cysts based on the ITS1-5.8S-ITS2 sequences and the GTR+I+G nucleotide evolution model, applying the consensus 50% majority rule.

Phylogenetic relationships of the found fungal species are presented as ML and BI dendrograms in the Figure 3 and Figure 4.

The results revealed that the mycobiota isolated from *H. filipjevi* cysts are represented by diverse taxa. Both ML and BI dendrograms are in agreement and generated the same clades. The two main clades corresponding to the phyla Ascomycota and Basidiomycota are separated by the Mucoromycota clade connected to the order Cantharellales, the order of uncertain taxonomic position. The frequency of Ascomycota within the total number of cultured cysts was 20.5% with *Pochonia* as the most prevalent species, while the frequency of Basidiomycota was 50% with *Bjerkandera* spp. as the most common basidiomycetous fungi. The least percentage pertains to Mucoromycota and *Linnemannia* species (2%). Ascomycota occurred in plots with intensive agricultural production, while Basidiomycota was more related to small-scale producers.

The Ascomycota clade consists of four subclades representing the families Clavicipitaceae with *Pochonia chlamydosporia*, Sarocladiaceae, and the representative *Sarocladium kiliense*, and the family Nectriaceae with *Fusarium avenaceum*. The families belong to the order Hypocreales. A subclade of the family Phaeosphaeriaceae (*Setophoma terrestris*) of the order Pleosporales is linked to the latter. The Basidiomycota clade is comprised of five subclades represented by the families Cerrenaceae (*Cerrena unicolor*), Polyporaceae (*Trametes hirsuta*), polyphyletic Phanerochaetaceae (*Bjerkandera adusta* and *B. albocinerea*) and a distinct subclade with *Phlebiopsis* spp., and the family Meruliaceae (*Phlebia/Mycoacia* spp.), all affiliated to the order Polyporales and the class Agaricomycetes. 

*Pochonia chlamydosporia* is a commonly found egg parasite in nematode suppressive soils. The fungus can remain saprotrophic in soil in the absence of both plant and nematode hosts. *Pochonia* spp. are found to be endophytes in some Gramineae and Solanaceae species colonizing the roots [27]. *Pochonia chlamydosporia* was found to produce phosphatases, enzymes that can degrade organic phosphate compounds. In addition, the fungus was able to solubilize inorganic phosphate and produce acetic, citric, and propionic acids [28]. Citric acid also extracted from *Aspergillus candidus* and a citric acid standard, each tested at 50 mg mL^−1^ in water, decreased egg hatching of second-stage juveniles of *Meloidogyne incognita* by more than 94% [29]. *Pochonia chlamydosporia* was found to parasitize eggs of the beet cyst nematode *H. schachtii* [30]. *Heterodera filipjevi* and *H. schachtii* were frequently found in mixed populations [31] and apparently have the same fungal parasites.

*Acremonium kiliense* and *A. zeae* were transferred to the genus *Sarocladium*, phylogenetically distinct from the *Acremonium strictum* clade, according to the combined SSU/LSU analysis [32]. Several species of the genera *Acremonium* and *Sarocladium* caused brown spots on bagged apples [33]. *Sarocladium kiliense* was found to possess both antifungal and antinematode properties. Treatment of the leaf pieces with *Sarocladium kiliense* conidia for one or three days prior to inoculation with *Diaporthe (*syn. *Phomopsis) longicolla*, a seedborne fungal disease that causes yield losses and reduced seed quality of soybean, eliminated pycnidial development completely [34]. Methanol extracts from mycelium of *Sarocladium kiliense* (0.3 mg mL^−1^) and fungal culture filtrate (1 mL) induced 35–37% mortality of *Meloidogyne incognita* second-stage juveniles [35].

Fungi of the genus *Fusarium* are worldwide pathogens of cereals. The metabolites produced by *Fusarium avenaceum* include: moniliformin, beauvericin, enniatins, chlamydosporols, chrysogine, acetamido-butenolide, antibiotic Y, fusarins, aurofusarin, etc. [36]. *Fusarium avenaceum* reduced wheat yield up to 25% in a field experiment in Switzerland [37]. *Fusarium* spp. caused 34–52% mortality of *M. incognita* second-stage juveniles in in vitro studies [38].

*Setophoma terrestris* is designated as one of the most serious pathogens in tropical and subtropical soils [39]. Among several tested fungi, *Setophoma terrestris* were shown to decompose various glucosinolates [40]. *Pyrenochaeta (*syn*. Setophoma) terrestris* reduced 60% egg hatching of second-stage juveniles of the soybean cyst nematode *H. glycines* [41].

The members of the order Polyporales of the Basidiomycota clade belong to the white rot fungi or wood-decay fungi and represent a source for prospective novel producers and novel compounds [42] and also important agents for bioremediation.

The molecular phylogenetic analysis of white rot fungi, confirmed that the genera *Mycoacia* and *Mycoaciella*, as well as *Merulius*, should be considered as synonyms of *Phlebia* [43]. In this study, *Phlebia* was nested within two previously named *Mycoacia* species in both dendrograms. The white rot fungus *Phlebia* sp. MG-60 produced ethanol directly from cellulose, glucose, and xylose, and could be considered a promising bioprocessing agent in biomass fermentation [44].

*Phlebiopsis gigantea* invades the sapwood and degrades resin and other wood extractives, demonstrating that the fungus is an ideal candidate for use in biological processing. It was found that *P. gigantea*, when applied to cut stumps, could inhibit subsequent colonization by the pathogen *Heterobasidion annosum*, a root rot fungus [45]. *Phlebia* and *Phlebiopsis* species were not able to infect or destroy *Aphelenchoides* spp. [46], nematodes that usually inhabit aerial parts of plants.

*Bjerkandera adusta* and its sister species *B. albocinerea* species were originally described from temperate Europe and Brazil, respectively, growing mainly on dead deciduous hardwood logs [47]. Polycyclic aromatic hydrocarbons are high-risk pollutants that affect human health because of their carcinogenic and mutagenic effects. It has been proposed that ligninolytic enzymes are key enzymes in the degradation of benzopyrene by *B. adusta* SM46, which suggests its bioattenuation and bioremediation potential [48]. *Bjerkandera adusta* strain had low activity against juveniles of the nematode *Steinernema carpocapsae* [49].

*Trametes hirsuta* MTCC-1171 could use ferulic acid as a sole carbon source. Ferulic acid is being considered as an environmental pollutant, since wine distilleries, oil, and paper processing industries produce effluents containing ferulic acid [50]. *Trametes versicolor*, performing as a plant growth promoter, exhibited an increase in wheat grain yield of 37%, as well as straw yield of 27% as compared to non-colonized plants [51]. *Trametes trogii* cultured on the glucose-peptone agar showed low activity against juveniles of the nematode *S. carpocapsae* [49].

*Cerrena unicolor* produces laccases, copper-containing oxidoreductive enzymes, which reduce oxygen to water and, typically, oxidize a phenolic substrate demonstrating its suitability for environmental detoxification [52]. The other species, *Cerrena (*syn. *Trametes) maxima*, has the potential to degrade the herbicide atrazine [53].

Lichenicolous fungi, such as *Burgoa* spp., are a highly specialized and successful group of organisms that develop on lichens and form numerous ecological associations with them [54]. Lichens are ubiquitous organisms that inhabit even extreme environments e.g., Antarctica [55]. The basidiomycetous, bulbilliferous *Burgoa* spp. were isolated from *Populus* wood [56], biodeteriorated murals, plaster, and stone walls [57]. In this study, *Burgoa verzuoliana* was phylogenetically placed as the closest taxon to *Mortierella/Linnemannia* as a distinct clade being genetically closer to Basidiomycota.

*Mortierella* i.e., *Linnemannia*, a cosmopolitan soil fungus, was found to possess numerous biodegradation abilities. Recent studies have shown that *M. elongata* isolated from *Populus* is able to promote its growth. *Mortierella elongata* isolates PMI 624 and PMI 93 increased the plant height, leaf area, and plant dry weight of watermelon, maize, tomato,andsquash. *Mortierella* had a significant role in soil carbon and phosphorous cycling, and chitin degradation [58], in increasing the levels of plant indole acetic acid and plant biomass [59], and in degradation of volatile compounds from different hydrocarbon fuels [60]. Among various microorganisms screened for arachidonic acid productivity, a precursor of prostaglandin, involved in inflammatory processes [61], an isolated fungus identified as *Mortierella elongata* strain IS-5, was found to show the highest productivity [62]. To stabilize Mortierellaceae taxonomy the genus *Linnemannia* was erected to include the monophyletic *gamsii* clade, which contains the *L. elongata* complex, *L. gamsii*, *L. amoeboidea*, and related species [63]. *Mortierella globalpina* was demonstrated to prey upon *Meloidogyne chitwoodi* by adhering a fungal hypha to the nematode cuticle and consequently consume the nematode [64].

Among wood-decay fungi, there is a host preference between gymnosperms and angiosperms. *Bjerkandera adusta*, *Cerrena unicolor*, *Fomes fomentarius, Irpex lacteus*, *Trametes hirsuta*, and *T. versicolor* were exclusively found on angiosperms. Birch, poplar, and willow trees were the preferential hosts for *Bjerkandera adusta*, *Cerrena unicolor*, *Phlebia* spp. and*Trametes hirsuta* [65], the fungal species also found in this study and reported as endophytes from multiple hosts [66]. There is a lack of available nitrogen in wood and, therefore, nematophagous fungi (which showed good ability to colonize wood) satisfy their nitrogen requirements by capturing nematodes [7]. Endophytes can produce the same or similar secondary metabolites as their host plants. The endophytic fungus *Taxomyces andreanae* produced the same compound-Taxol as its host *Taxus brevifolia* [67]. The fungal endophyte of the cinnamon (*Cinnamomum zeylanicum*), *Muscodor albus* (syn. *Induratia alba*) was found to produce volatile antimicrobial compounds with bactericidal and fungicidal properties [68]. Since it has recently been found that *Cinnamomum cassia* and *C. burmanii* essential oils have the highest nematicidal activity on a psychrophilic panagrolaimid nematode [69], probably the same fungal endophyte would have a similar nematicidal effect.

It was not unexpected that the plant parasitic fungi might occur in crops and subsequently they could be transferred to soil. In contrast, the species of the phylum Basidiomycota (*Phlebia nothofagi*, *Phlebiopsis ravenelii*, *Bjerkandera adusta*, *Trametes hirsuta*, and *Cerrena unicolor*) were reported to be associated with birch, poplar, and willow forest ecosystems [65], but their presence was surprising in agricultural soil of the Pannonian Plain. The explanation was found through the analysis of historical data.

Several authors of the Principate period of the Roman Empire testify to the fact that the Roman province of Pannonia was a densely wooded area. Classical authors refer to a whole range of different species used: oak, beech, fir, hazel, ash, alder, as well as different types of willow [70]. The forests of Slavonia and Srem (The Southern Pannonia) were also described by the Austrian subjects Friedrich Wilhelm von Taube and Franz Stefan Engel, in the second half of the 18th century [71]. In order to protect the Serbian natural values in this area, the Institute for Nature Conservation of Serbia designated two zones mostly covered by willow and poplar forests and Canadian poplar plantations [72]. Recently, the two localities of the ancient beech forests in Fruska gora were included in Europe world heritage sites, witnessing the presence of beeches in the ancient Pannonian Plain [73]. Ancient and modern historical data indicate that forests, especially deciduous forests were the preceding plants before the Pannonian Plain was turned into arable land. The area was occupied with beech, birch, oak, poplar, and willow trees that were typical hosts of the found basidiomycetous species. Yet there still exist scattered deciduous forests and trees in the vicinity of the studied localities.

## 4. Conclusions

Regarding the higher fungal taxonomy, the study showed that isolated fungal species belong to diverse phyla, such as Ascomycota, Basidiomycota, and Mucoromycota. The phylum Ascomycota is divided into the order Hypocreales, represented by the families Clavicipitaceae, Sarocladiaceae, and Nectriaceae, and the order Pleosporales, represented by the family Phaeosphaeriaceae. The phylum Basidiomycota is divided into the order Polyporales, represented by the families Cerrenaceae, Polyporaceae, Phanerochaetaceae, and Meruliaceae, and the order Cantharellales of uncertain taxonomic position, but phylogenetically affiliated to the Basidiomycota clade. The phylum Mucoromycota is linked to the order Cantharellales and also phylogenetically closer to Basidiomycota than to Ascomycota. Most of Basidiomycota are wood-decay fungi with a great enzymatic potential for bioremediation in polluted environments. The isolated basidiomycetous species have a host preference towards deciduous trees, such as birch, poplar, and willow trees, historical data of which confirmed that in the Pannonian Plain massive deforestation occurred during centuries, turning forest land into arable land. The members of Ascomycota are plant and nematode parasites, indicating that crops were host plants for fungal infection of recent origin. The members of Basidiomycota are wood-decay fungi, the presence of which in agricultural soil indicates that forests were the preceding plants enabling the plant field colonization history to be traced chronologically. 

## Data Availability

Not applicable.
